# Who has mental health problems? Comparing individual, social and psychiatric constructions of mental health

**DOI:** 10.1007/s00127-023-02474-4

**Published:** 2023-04-17

**Authors:** Bernice A. Pescosolido, Harold D. Green

**Affiliations:** 1 Department of Sociology, College of Arts & Sciences and the Irsay Institute, Indiana University, IN Bloomington, USA; 2 Department of Applied Health, School of Public Health and the Irsay Institute, Indiana University, IN Bloomington, USA

**Keywords:** Social construction, Unmet need, Labelling theory, Mental health literacy

## Abstract

**Purpose:**

The persistent gap between population indicators of poor mental health and the uptake of services raises questions about similarities and differences between social and medical/psychiatric constructions. Rarely do studies have assessments from different perspectives to examine whether and how lay individuals and professionals diverge.

**Methods:**

Data from the Person-to-Person Health Interview Study (P2P), a representative U.S. state sample (N ~ 2700) are used to examine the overlap and correlates of three diverse perspectives—self-reported mental health, a self/other problem recognition, and the CAT-MH™ a validated, computer adaptive test for psychopathology screening. Descriptive and multinominal logit analyses compare the presence of mental health problems across stakeholders and their association with respondents’ sociodemographic characteristics.

**Results:**

Analyses reveal a set of socially constructed patterns. Two convergent patterns indicate whether there is (6.9%, The “Sick”) or is not (64.6%, The “Well”) a problem. The “Unmet Needers” (8.7%) indicates that neither respondents nor those around them recognize a problem identified by the screener. Two patterns indicate clinical need where either respondents (The “Self Deniers”, 2.9%) or others (The “Network Deniers”, 6.0%) do not. Patterns where the diagnostic indicator does not suggest a problem include The “Worried Well” (4.9%) where only the respondent does, The “Network Coerced” (4.6%) where only others do, and The “Prodromal” (1.4%) where both self and others do. Education, gender, race, and age are associated with social constructions of mental health problems.

**Conclusions:**

The implications of these results hold the potential to improve our understanding of unmet need, mental health literacy, stigma, and treatment resistance.

“Disease is a social entity, not an array of ideal types.” *Charles Rosenberg* [1:237]

## Introduction

Perennial concerns among mental health providers, consumers, advocates, and policymakers surround the mismatch between epidemiologically derived levels of population mental illness and correspondingly low levels of treatment [2–4]. While diverse reasons, including geographic inaccessibility and financial costs, are implicated in this gap, mental health literacy targets deficits in knowledge among the public to recognize mental illness and know what to do in response [5]. From social construction and labelling perspectives, others caution that psychiatry and medicine may see cultural difference as “disease” [6, 7]. While solid bodies of research exist on the social construction of clinical diagnoses (e.g., [8–10]) and lay diagnoses (e.g., [11]), what is missing are explicit efforts comparing how constructions differ.

Such differences tap into debates on the meaning and definition of mental illness. From controversies within psychiatry about the nature and categorization of mental illness in diagnostic categories [8, 9, 12, 13] to the more radical labelling critiques of the medicalization of non-normative behavior [6, 14], the identification of mental illness has and continues to face serious challenges in separating problems of daily living and clinically relevant problems. Similarly, advocacy groups and others question the biomedical approach, arguing for greater reliance on quality of life, recovery and acceptance of non-neurotypical minds rather than symptoms and their reduction (e.g., [15, 16]). Discrepancies are further fueled by the dramatic cultural transformation of mental health and illness from a niche concern among specialists practicing in secluded institutions to a contemporary public health crisis fostered by a persistent lack of community services; a documented rise in youth mental illness and suicides; widespread recognition of mental health disparities for racial, ethnic and poor populations; and the dramatic mental health burden brought by the global COVID-19 pandemic [17–20]. Coupled with the increase in pharmaceutical advertising, public health campaigns, and all forms and valences of media attention, marking the convergence and divergence among public and professional constructions holds the potential to offer insights into understanding issues of unmet need, mental health literacy, medicalization, stigma, and treatment resistance. Yet, despite perpetual debates on understanding how and for whom views of mental health problems differ, research on discrepancies among stakeholders remains relatively rare.

According to medical historian Charles Rosenberg [21], the modern professional construction of mental health and illness has focused primarily on the issue of diagnosis. He, among others (e.g., [22, 23]), have described the shift from “dynamic psychiatry” where providers in the Freudian tradition conceptualized mental health problems as broad, continuous, and diffuse to “diagnostic psychiatry” where, in the Kraepelin tradition, psychiatrists categorized mental health problems as discrete disease entities. The latter were eventually codified into the various versions of the Diagnostic and Statistical Manual (DSM, [24]). This development, however, did not end controversy. Those who embrace the DSM describe how developing a common language among clinicians, researchers, health insurers, and the pharmaceutical industry replaced the “tower of Babel” that marked the pre-DSM era (e.g., [13, 25, 26]). They argue that the cut-off between “normal” and “pathological” encourages collaboration and improves treatment. A wide range of critics, within and outside of psychiatry, responded that “an ever-broader variety of emotional pain, idiosyncrasy, and culturally unsettling behaviors” has been translated into a continually expanding disease list [21:407]. This “diagnostic inflation” [9] is evidenced in specific battles fought for or against homosexuality, premenstrual syndromes, bereavement, and ADHD as mental disorders [27–33]. Even the attempt to move to DSM-5 Task Force’s suggested dimensional system was criticized as premature by psychiatrists and stakeholders [34, 35].

As Jutel [10] points out, diagnosis is a powerful social tool which takes place at a critical interaction between people and providers; communities and medical systems; and complaints expressed and explanations offered. In this nexus, the social construction of mental health problems becomes central. In extreme versions, both psychiatrists and social scientists originally proffered ideas that mental illness is simply a myth, a label applied to those whose behavior falls outside norms, a self-fulfilling prophecy, and an instrument of social control [6, 12, 36]. More recent arguments view mental illness as real but caution that psychiatric “naming and framing” efforts [37] to harness mental health problems into discreet categories is neither clearly evidence-based, neutral, nor inconsequential for individual, families, or societies. The DSM is seen as insensitive to cultural differences in “normal” or even prevailing behavioral norms. It embeds and reflects existing prejudices and stereotypes which place certain constellations of identities at greater risk for being (mis)diagnosed [38–40]. Black men are more likely to be diagnosed as “schizophrenic” [7]; women with clear signs of heart disease are more likely to receive a depression rather than a cardiac diagnosis, [41]; and minority youth are more likely to be misdiagnosed in general [42]. In sum, age, race/ethnicity, gender, rural/urban residence, and education have been implicated consistently in defining the social and cultural vectors that shape individuals’ reaction to health, illness and disease.

Not surprisingly, medical sociologist Mildred Blaxter [43] initially conceptualized diagnosis as complex social phenomena, deeply embedded in history, science, and social conditions. The social construction of diagnosis [10] extended beyond the clinical to the community to include the concept of lay diagnosis. While clinical diagnoses mark “disease”; lay diagnoses mark “illness”, the social experience of changes in personal or social function that take shape in the community. Clinical diagnoses confer legitimacy on illness; but, as Jutel [44] points out, lay and psychiatric diagnoses may or may not align. These insights have been encoded in standard, individually based theories of utilization as problem recognition because they provide the precondition for decisions about service use. Freidson [45:286] goes one step further, arguing that lay constructions of illness are not simply an individual matter because others comprise a “lay referral system,” a centerpoint in the Network Episode Model [46–48]. Whether the individual or the social network is considered, research documents the key role of lay diagnosis in service use patterns which, itself, may be shaped by sociodemographic and sociocultural conditions [11, 49–52].

The benefit of multiple assessments of mental health (individual assessments of overall mental health, a socially based self-reflection of mental health problems, and a more clinical diagnostic assessment of mental health) is that we move beyond a one-dimensional perspective to one that explores definitions of mental health across three axes. In many ways this is similar to newer approaches that explore a ‘dual’ continua model of mental health where the diagnosis or existence of a mental health condition is not necessarily linked to a negative assessment of that condition or of overall mental health [53]. This is our perspective. In fact, beginning with Anthony’s (1993 [54]) “guiding vision” of recovery, rather than symptom reduction as the measure of success, even for people with serious and persistent mental illness, the question of what and whose assessment matters has been called into question [53, 55].

Here, we draw from a representative, statewide omnibus health survey, the Person-to-Person Health Interview Study (P2P) to compare the nature, overlap and correlates of constructions of mental health problems from three distinct perspectives—the self, the social network, and the psychiatric profession. The first, most common and simplest measure, relies on individuals’ self-assessment of their mental health. The second measure asks respondents whether they or those around them suggested that they have a mental health problem. The third deploys a state-of-the-art psychiatric epidemiology measure, the CAT-MH™, a distinct suite of validated, computer adaptive tests for psychopathology. While the scientific base to develop hypotheses is slim and contradictory, the public health concern with unmet need suggests that the public, whether the individual or those around them, is less likely to recognize a mental health problem compared to clinical diagnostic measures. The sociological and anthropological perspectives suggest that efforts to medicalize problems of daily living would trickle down through mass advertising, especially as the internet has become a source of self-diagnosis [56]. As a result, the public may see more mental health problems, particularly for certain groups, than clinical standards would. We step back and ask three basic questions to lend evidence to adjudicate these diverse expectations: How do individual, social network, and professional assessments of mental health problems overlap? What are the patterns of convergence and divergence? And are patterns associated with sociodemographic or other contingencies?

## Methods

### Data source and study sample

The Person-to-Person Health Interview Study (P2P) is an omnibus health and wellness study based on face-to-face interviews. Designed to study multilevel factors that shape health, P2P used a stratified probability sample of households across the state of Indiana (US). Interviews were conducted with a target random sample of 2700 residents of Indiana, representative with respect to age, ethnicity, urbanicity, and gender, from October 23, 2018, to March 21, 2020, when interviewers were pulled from the field to protect them and respondents as the COVID-19 pandemic became prevalent in Indiana. Interviews resumed from July 16, 2020, to June 30, 2021 (N = 2685). After deletion of respondents with missing age, race, sex, number of total adults in the household, or key mental health outcomes, the effective sample for this analysis is N = 2559 individuals. These data avoid the limitations of surveys using convenience samples or respondent panels, which are vulnerable to response and selection bias, particularly limiting inclusion of people with lower incomes, those with less education, and those living in rural communities. Sample and weights were provided by NORC.

### Measures

#### Mental health outcomes

Three different mental health outcomes come from three separate P2P modules. The individual construction of mental health is based on a self-assessment using the following question “Would you say that, in general, your mental health is…” This general question from the individual health behavior module allows respondents to work from their own cognitive framework. Based on response distributions, categories of excellent, very good, good, fair, and poor were dichotomized with fair and poor mental health (coded 1; 0 otherwise). The social network construction of mental health problems/mental illness is taken from a question in a module focused on social connections and contexts. It asked respondents: “During the last year, have you thought or has someone told you that they thought that you might have a mental health or emotional problem?” with yes (coded 1) and no (coded 0) as response options. The professional construction of population-based diagnosis used the CAT-MH™ diagnostic battery. The assessment takes a computer adaptive approach to assess a range of mental illness diagnoses with questions pulled from a bank of over 3,000 items [57]. The clinical assessment combines the results from the mental health test batteries for “Major Depressive Disorder” (where diagnosis = positive), “Depression” (where severity = moderate or severe), “Anxiety Disorder” (where severity = moderate or severe), and “Mania/Hypomania” (where severity = moderate or severe). Respondents were coded as having (1) or not having (0) a mental health diagnosis.

Table 1 provides the profile of respondents used here. Comparison with the U.S. Census data for Indiana [58] indicates that P2P data align broadly with State population statistics. On the dependent variables, nearly one fifth (19.2%) of respondents rated their own mental health as fair or poor. Only 15.8% reported that someone they know had told the respondent that they felt he/she/they may have a mental health or emotional problem. Nearly one quarter (24.5%) met the criteria for a clinical diagnosis for Major Depressive Disorder or moderate to severe depression, anxiety, or mania/hypomania. This last statistic is slightly higher than the 21% national prevalence reported by the National Institute of Mental Health [59].

**Table 1:** Mental health status and sociodemographic characteristics, Person-to-Person Health Interview Study, 2018–2021 (N = 2559, unweighted)

	Overall N (%)
Self assessment: fair (14.3%) or poor (4.85%) mental health	491 (19.2%)
Clinical assessment: CAT-MH diagnosis	626 (24.5%)
Social assessment: mental health or emotional problems by you or others	403 (15.8%)
Age	
Average (std. dev)	50.6 (18.4)
18– < 35 years	649 (25.4%)
35– < 50 years	614 (24.0%)
50– < 65 years	626 (24.5%)
65 years and older	670 (26.2%)
Race	
Minority^1^	346 (13.5%)
White	2213 (86.5%)
Gender	
Male	972 (38.0%)
Female	1575 (61.6%)
Transgender^2^	11 (0.4%)
Education	
< HS	213 (8.3%)
HS or GED	676 (26.4%)
Some college	601 (23.5%)
Technical certificate/associates degree	332 (13.0%)
College degree	737 (28.8%)
Rurality	
Urban	1828 (71.4%)
Rural	731 (28.6%)

^1^Categories were Hispanic/Latino, Non-Hispanic Black/African American, Non-Hispanic Asian, Non-Hispanic American Indian, Native Hawaiian/Pacific Islander, and other/Non-Hispanic multiple races

^2^The transgender category presented in this table includes those who identified as transgender, non-binary or gender fluid, genderqueer, intersex, or any other non-cis gender identities

#### Sociodemographic variables

Rural status was assigned based on county of residence. Using NORC standards, counties that had a Metropolitan status were defined as urban, rural otherwise. As indicated in Table 1, respondents were overwhelmingly from urban areas (over 70%), which aligns with the 2020 U.S. Census estimate for Indiana of 66.5%. Race/ethnicity was based on respondents’ self-identification based on standard U.S. Census categories and dichotomized into white (86.5%) versus other, aligning with the most current U.S. Census estimate for Indiana (84.8%). Respondents were asked to self-identify sex and gender and recoded into three categories: male, female, other identities (including transgender, non-binary/gender fluid). Respondents were predominantly female (61.6%). With less than half of one percent reporting an alternative gender identity, gender was coded as male/female; 11 transgender individuals were dropped from the analysis. Respondent age was calculated as the difference between date of interview and birthdate. Respondents were evenly distributed with respect to age, with the average age being about 50 years old and the group percentages ranging from the lowest of 24.0% for those aged 25–50 and the highest of 26.2% for those 65 and older. Education was recorded as the highest level of education completed and coded into five categories: less than high school, high school graduate or GED, some college (no degree), technical certificates/associates degree, and college or higher. Over 90% of respondents have earned the equivalent of a high-school diploma or higher, with the largest proportion of respondents (28.8%) reporting having earned a college degree and 13.0% reporting having a technical or associate degree. Other cultural factors (e.g., migration status, 3.7%) were considered but of too low prevalence to include without jeopardizing coefficient stability.

### Statistical analysis

Descriptive statistics and graphics are presented to compare different perspectives of mental health status and demographic variables for the overall analytic sample. Specifically, on social construction differences, a Venn diagram using unweighted data provides a detailed examination of convergence and divergence across measures of mental health. Multinomial regression provides estimates of the association between Venn categories and five demographic variables listed above. Significance levels were set for two-tailed tests at p ≤ 0.05.

## Results

### Patterns of the social construction of mental health problems

**Fig. 1:**
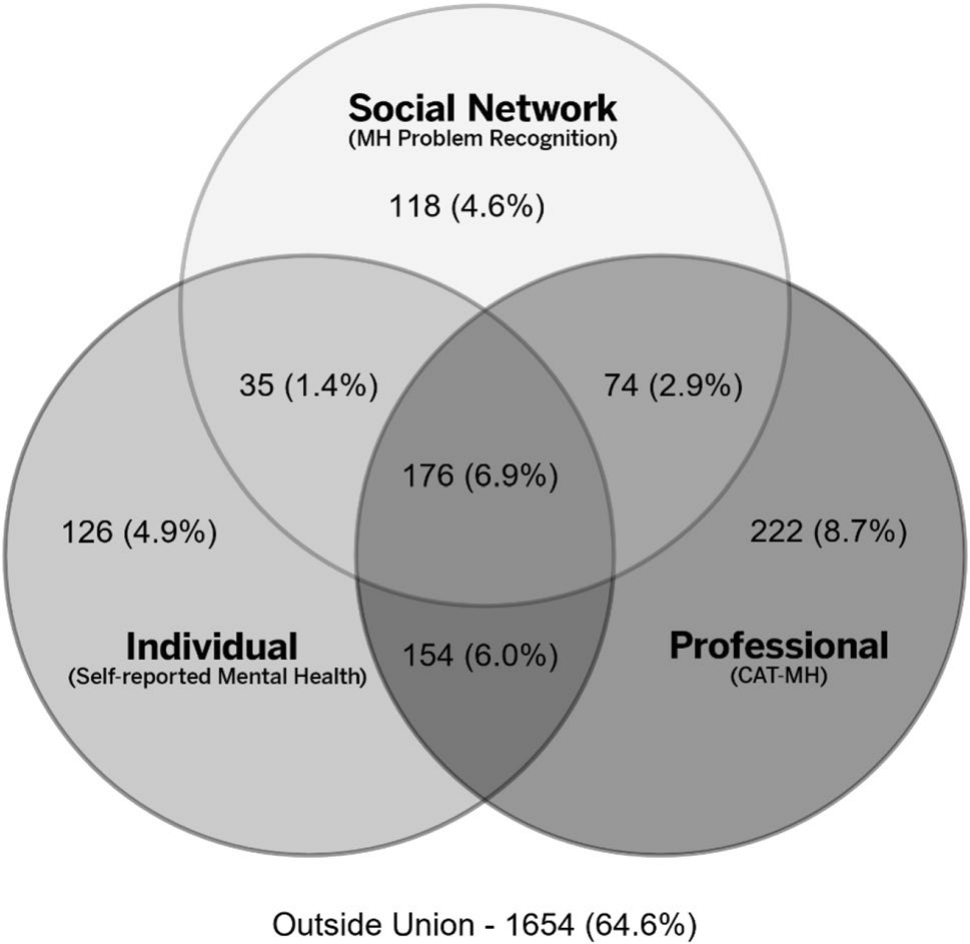
Convergence and divergence among social construction of mental illness, Person-to-Person Health Interview Study, 2018–2021 (N = 2559)

The overlap among the three mental health variables is shown in Fig. 1 and described in Table 2. The largest group of respondents, The “Well” (64.6%), falls outside the union of the three measures, indicating a large measure of agreement across self, social network and professional assessments of mental health problems. The remaining third of respondents reported “need” on at least one measure. Convergence across all three measures comprise a group labelled The “Sick” (6.9%). They, their social networks, and the diagnostic threshold all indicated a mental health problem. This percentage is strikingly close to the US estimate for serious mental illness in one year (5.6%) but much lower than “any diagnosed mental illness” in one year (21.0% [59]).

**Table 2:** Categorization of the patterns of convergence and divergence in self, social network and medical professional perspectives on individuals’ mental health status, Person-to-Person Health Interview Study, 2018–2021 (N = 2559, unweighted)

	The “Well”	The “Sick”	The “Unmet Needers”	The “Self Deniers”	The “Network Deniers”	The “Worried Well”	The “Network Coerced”	The “Prodromal”
Self construction: fair/poor MH	No	Yes	No	No	Yes	Yes	No	Yes
Clinical construction: CAT-MH	No	Yes	Yes	Yes	Yes	No	No	No
Social network construction: MH/emotional problem	No	Yes	No	Yes	No	No	Yes	Yes
Overall frequency % (N = 2559)	64.6% (1654)	6.9% (176)	8.7% (222)	2.9% (74)	6.0% (154)	4.9% (126)	4.6% (118)	1.4% (35)
Frequency within each category excluding the ‘well” % N = 905	NA	19.5%	24.5%	8.2%	17.0%	13.9%	13.0%	3.9%

One group met the thresholds for a CAT-MH™ diagnosis; however, neither they nor their social network members reported a problem. These are The “Unmet Needers” (8.7%) who, from the individual and public health perspectives, meet clinical criteria but are unrecognized in the community. The “Self Deniers” (2.9%) do not report poor or fair mental health themselves but are seen by both their social networks and identified by the CAT-MH™ as meeting criteria. The “Network Deniers” (6.0%) report that they have a mental health problem which is supported by the CAT-MH™ assessment but denied by their social networks. The “Worried Well” (4.9%) report poor mental health but neither their social network nor the professional construction support that view. Nearly the same number of respondents are The “Network Coerced” (4.6%) who are at risk for encountering pressure to seek out mental health care by their social network when neither they nor the CAT-MH™ identify a problem. Like some of the individuals in the Alang and McAlpine study [60] they may follow a ‘coerced’ pathway to mental health care but fail to meet diagnostic criteria if they do. Finally, only a small percentage of individuals fall into The “Prodromal” (1.4%) where the community constructions of self and social network see a problem but no clinically-relevant condition is detected at this point by the CAT-MH™.

### Correlates of the construction of mental health problems

These eight patterns are used to examine the association with socio-demographic characteristics. Table 3 reports the overall tests of significance across social construction patterns of mental health. Age, gender, and education discriminate across all the patterns while neither minority nor rural status do so.

**Table 3:** Overall statistical associations of socio-demographic characteristics on social construction patterns of mental health status, Person-to-Person Health Interview Study, 2018–2021 (N = 2559) al regressions

Effect	DF	Wald Chi-square	P value
Age	7	179.4660	< 0.001
Gender	7	26.0025	< 0.001
Education	28	114.6833	< 0.001
Race	7	8.1420	0.3202
Rural	7	5.8988	0.5516

A more fine-grained analysis of the effects of correlates on different social constructions requires sets of multinomial regressions where correlates are examined on each contrast of two pathways. Focusing on one contrast – The “Well” compared to the other seven categories indicates that older individuals are significantly more likely to be among The “Well” than any other category (Table 4). Women are more likely than men to be among The “Sick” or “Self Deniers” or “Network Coerced”. Those with less than a high school education (compared to some college) are more likely to be among The “Sick”, “Unmet Needers”, or find their claims of mental health problems denied by their networks or the care system (i.e., The “Network Deniers” or “Worried Well”). Conversely, those with at least a college degree (compared to some college) were less likely to find themselves among The “Sick”, “Unmet Needers”, and “Network Deniers”. Being a person of color has one effect. Compared to The “Well”, if they self-report a problem confirmed by the CAT-MH™, they are unlikely to be pressured into care by those around them. Rural residence did not distinguish among social construction patterns.

**Table 4:** Multinomial regression models predicting social construction patterns of mental health status, Person-to-Person Health Interview Study, 2018–2021, reference category is The “Well”, (N = 2559)

	The “Sick”	The “Unmet Needers”	The “Self Deniers”	The “Network Deniers”	The “Worried Well”	The “Network Coerced”	The “Prodromal”
AGE (older) Unit = 10 years	0.57*** (0.52, 0.64)	0.89** (0.82, 0.96)	0.67*** (0.58, 0.78)	0.85*** (0.78, 0.94)	0.90* (0.81, 1.00)	0.61*** (0.54, 0.69)	0.66*** (0.54, 0.82)
GENDER Ref = male	1.79** (1.25, 2.55)	1.02 (0.76, 1.36)	1.92* (1.13, 3.25)	0.96 (0.68, 1.35)	1.12 (0.77, 1.63)	2.09*** (1.35, 3.22)	0.76 (0.39, 1.50)
EDUCATION Ref = High school graduate/GED Less than high school	2.34** (1.35, 4.07)	2.51*** (1.56, 4.05)	2.57* (1.06, 6.27)	3.08*** (1.82, 5.20)	1.83 (0.98, 3.42)	1.03 (0.38, 2.80)	2.12 (0.64, 7.00)
Some college (no degree)	0.74 (0.48, 1.13)	0.99 (0.68, 1.46)	1.14 (0.57, 2.24)	0.94 (0.59, 1.48)	0.73 (0.44, 1.20)	0.93 (0.53, 1.64)	0.63 (0.23, 1.68)
Tech certificates/Assoc. degree	0.63 (0.36, 1.10)	0.67 (0.4, 1.11)	0.99 (0.43, 2.30)	0.75 (0.42, 1.33)	0. 70 (0.39, 1.28)	1.16 (0.61, 2.21)	0.76 (0.23, 2.46)
College or higher degree	0.29*** (0.18, 0.49)	0.49*** (0.32, 0.74)	0.93 (0.47, 1.82)	0.42*** (0.25, 0.70)	0.45** (0.27, 0.76)	1.07 (0.64, 1.81)	0.68 (0.28, 1.67)
RACE person of color Ref = white	0.76 (0.47, 1.21)	1.02 (0.67, 1.54)	0.72 (0.35, 1.46)	0.82 (0.49, 1.38)	0.90 (0.52, 1.56)	0.44* (0.23, 0.84)	0.73 (0.27, 1.96)
URBAN Ref = rural	0.91 (0.64, 1.31)	0.84 (0.62, 1.15)	0.96 (0.56, 1.63)	0.72 (0.50, 1.04)	0.97 (0.64, 1.46)	1.22 (0.78, 1.92)	1.38 (0.61, 3.13)

Bold values and asterisks indicate the significance of correlates (age, gender, education, race, urbanicity) in predicting the seven social construction patterns of mental health. * *p* < 0.05, ** *p* < 0.01, *** *p* < 0.001

**Fig. 2:**
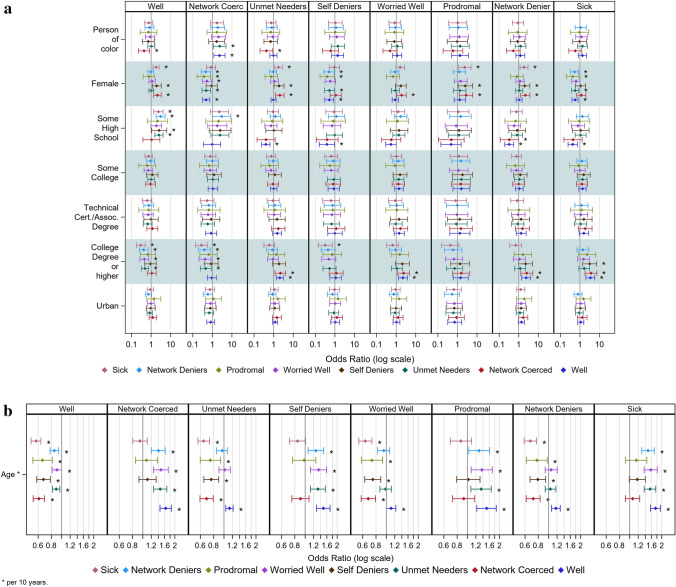
**a** Odds ratios and standard errors for multinomial regression models across all reference categories, binary variables. Relevant reference category is indicated by column name. Significant effects are noted with an asterisk, Person-to-Person Health Interview Study, 2018–2021. **b** Odds ratios and standard errors for multinomial regression models across all reference categories, continuous variables. Relevant reference category is indicated by color. Significant effects are noted with an asterisk, Person-to-Person Health Interview Study, 2018–2021

The graphical summary of effects across all social construction categories, representing sets of multinomial regressions, presents the odds ratios and confidence intervals (Fig. 2a and b). Both more detailed and relatively complicated, they are interpreted as follows. Each column represents a reference category in a multivariate regression that examines the effect of independent variables on whether individuals are more or less likely to be in that pattern than the reference category (the column title).

The upper left corner, Fig. 2a, represents seven analyses examining race effects in comparison to The “Well”. As noted above, people of color are less likely to be among The “Network Coerced” (leftmost cell; second cell from left, third cell from left) than The “Well” or “Unmet Needers”. The findings align with health disparities research that when people of color likely have a clinically diagnosable mental health problem, they are more likely to consider themselves well, more likely to have unmet need, and less likely to have social networks that facilitate help-seeking [20].

Women are more likely to be among The “Sick”, than most of the other categories (e.g., “Well”, “Unmet Needers”, “Prodromal” or “Network Deniers”). However, they are also more likely to be among The “Self-Deniers” than “Unmet Needers”, “Network Deniers” or “Prodromal”. These findings line up with research on women’s mental health (e.g., [61]), their greater reluctance to see themselves as having a problem, but more likely to seek care (at least initially [62–64]).

Those with lower levels of education (some high school relative to having a high school diploma or GED) are also more likely to be among The “Sick”, “Self-Deniers”, “Network Deniers” or “Unmet Needers” compared to The “Well”. They are also more likely to be in The “Network Deniers” than “Network Coerced”. Epidemiological studies consistently document high levels of mental health problems combined with low levels of use in this group [3, 65]. These findings suggest that, despite indicators of need, that individuals may be unable to recognize mental health problems; and even if they do, that those around them may tend to deny the problem [66, 67]. Those with advanced education are more likely to be among The “Well” and The “Network Coerced” than The “Sick”, “Network Deniers”, “Worried Well” or “Unmet Needers”. They are also more likely to the among The “Self-Deniers” than The “Sick.” For those with college education, the social construction of lay, network and professional groups all come together to reflect better mental health of those with higher status. In addition, their higher documented use of service not only reflects their own recognition of problematic versus non-problematic mental health; but, if they deny problems (although they are significantly less likely to be deniers), their social networks are likely to provide a greater push into treatment, decreasing unmet need.

Older individuals are less likely to be in any other profile than The “Well” (leftmost cell, Fig. 2b). In line with this, they are more likely to be in most other profiles than The “Sick” or “Prodromal” (sixth cell). They are less likely to be pushed into care by their networks who see need when they don’t (second cell from the left) or to deny need (fourth cell from the left). However, if they perceive mental health problems, they may risk unmet need (third cell from the left) or be seen as among The “Worried Well” (fifth cell from the left).

## Discussion

We deployed representative, population data to examine how individuals’ perceptions of their own mental health status aligns with or diverges from their social networks or psychiatry’s diagnostic rules. The greatest alignment across all three perspectives, which may provide relief from sociomedical concerns, occurs among those individuals who do not have mental health problems. Almost two thirds of the sample show concordance of individual, social network, and psychiatric perspectives that their mental health is fine. While much lower (~ 9%), The “Unmet Needers” are the second highest category. Here, diagnostic criteria suggest need but neither individuals nor their networks agree. From a psychiatric perspective, programs of individual and community-based education continue to be necessary for such individuals to receive care. However, in absence of “signs” of mental illness (e.g., blood tests) and none on the horizon [13], overdiagnosis, over-medication, and stigma may result if the individual and social network perspectives better map the situation here. As noted earlier, those seen as having mental health problems from all perspectives comes close to US population estimate of serious mental illness in any one year but far away from NIMH prevalence figures [59]. Overall, The “Sick” are more likely to be younger, women, people of color, and with lower levels of education. This convergence of need reflects known epidemiological groups at risk for mental health problems, and for health care disparities.

The more complicated profiles suggest pockets of concern for either under or over diagnosis. On the positive side, findings suggest that as individuals age, they are less like to be at risk for or deny current/future mental health problems or experience coercion from their social networks. However, if older individuals are in The “Sick” category, they may be at risk for unmet need or to have problems denied by social networks or psychiatrists.

In more than a modest percentage of cases (~ 30%), there is a mismatch of perspectives. Here the concerns about whether and how to respond are more difficult, in part because there is no clear homogeneity of possible reasons nor clear courses of action. For one female subgroup, need is denied even when the social network and psychiatry say otherwise. Another subgroup of women may be coerced into care by their networks when neither they nor psychiatry see need. For youth, one subgroup is more likely to either deny need; another is part of The “Worried Well”, and a third is at risk for coercion into care. People of color are more likely to have unmet need, in one subgroup profile, because neither they nor those around them recognize psychiatrically defined problems. However, among a different subgroup, people of color are less likely to have unmet need than to be coerced into care by those around them, putting them at risk for service use that does not result in a diagnosis [60]. Among another subgroup, claims of need by people of color for care are rejected by both the health care system and their social networks. As a final example, while those with the lowest levels of education are more likely to be among The “Sick”, they are both more and less likely to receive pressure from others around them and to have unmet need compared to those who have a high school degree.

Every study has limitations, and this study is no different. We believe that there are four key ones. First, our survey was developed through an iterative review and pilot testing process that allowed us to assess how respondents would interpret our questions so that we could adjust the language if necessary. Despite that process, for the two self-reported items used in our study there may have been some overlap between a respondent’s self-assessment of their mental health and their ‘social’ assessment of their mental health though none of our respondents or our interviewers mentioned issues with the questions. The presence of a trained interviewer to answer any questions during the survey and the clear difference in the various P2P modules should have been adequate to address any confusion. However, there remains the possibility that respondents answered the two questions similarly. Second, while our analyses explored the independent effects of key health factors such as age, race, gender, and education and found important associations, we did not explicitly or fully explore all possible meaningful interactions given the complexity of the number of patterns and multinomial logit analysis. Third, our categories are given labels that operate primarily to capture the clinical/medicalized meanings associated with the ‘unmet need’. Even as the most frequently used framework, different group names would be equally possible and useful. Finally, our analyses addressed mental health broadly, initially combining those who screen for depression, anxiety and mania/hypomania into a group of individuals with clinically detected mental health issues because this was more aligned with the broader individual and social conceptualizations of ‘mental health.’ Future analysis will address these limitations and explore these relationships in greater detail.

Despite possible limitations, looking at the question of “Who has a mental health problem?” from three different perspectives provides unique insights. The alignment of the percentage of individuals where all three perspectives converge tends to align with NIMH estimates. Further, the level of convergence among those with no mental health problems suggests that under or over utilization may be lower than commonly thought. However, where there is divergence, subgroups suggest different policies and programs because unmet need does not have the same source for youth, women, people of color, or those with low levels of education. Some profiles suggest that care should be provided, and barriers removed; others raise questions about whether treatment would help or hurt. While we cannot adjudicate the underlying reality, of course, investigating the source of discrepant views of mental health problems, especially in the clinical setting, may be required for developing tailored and effective practices and policies.

## Data Availability

The data that support the findings of this study are not publicly available at the time of publication. The data are, however, available from the authors upon reasonable request and with the permission of the Person-to-Person Health Interview Study Principal Investigators. To inquire about access to P2P data, please email irsay@iu.edu.
